# An Intertwined Evolutionary History of Methanogenic Archaea and Sulfate Reduction

**DOI:** 10.1371/journal.pone.0045313

**Published:** 2012-09-21

**Authors:** Dwi Susanti, Biswarup Mukhopadhyay

**Affiliations:** 1 Virginia Bioinformatics Institute, Virginia Tech, Blacksburg, Virginia, United States of America; 2 Genetics, Bioinformatics and Computational Biology Ph.D Program, Virginia Tech, Blacksburg, Virginia, United States of America; 3 Departments of Biochemistry, Virginia Tech, Blacksburg, Virginia, United States of America; 4 Biological Sciences, Virginia Tech, Blacksburg, Virginia, United States of America; The Centre for Research and Technology, Hellas, Greece

## Abstract

Hydrogenotrophic methanogenesis and dissimilatory sulfate reduction, two of the oldest energy conserving respiratory systems on Earth, apparently could not have evolved in the same host, as sulfite, an intermediate of sulfate reduction, inhibits methanogenesis. However, certain methanogenic archaea metabolize sulfite employing a deazaflavin cofactor (F_420_)-dependent sulfite reductase (Fsr) where N- and C-terminal halves (Fsr-N and Fsr-C) are homologs of F_420_H_2_ dehydrogenase and dissimilatory sulfite reductase (Dsr), respectively. From genome analysis we found that Fsr was likely assembled from freestanding Fsr-N homologs and Dsr-like proteins (Dsr-LP), both being abundant in methanogens. Dsr-LPs fell into two groups defined by following sequence features: Group I (simplest), carrying a coupled siroheme-[Fe_4_-S_4_] cluster and sulfite-binding Arg/Lys residues; Group III (most complex), with group I features, a Dsr-type peripheral [Fe_4_-S_4_] cluster and an additional [Fe_4_-S_4_] cluster. Group II Dsr-LPs with group I features and a Dsr-type peripheral [Fe_4_-S_4_] cluster were proposed as evolutionary intermediates. Group III is the precursor of Fsr-C. The freestanding Fsr-N homologs serve as F_420_H_2_ dehydrogenase unit of a putative novel glutamate synthase, previously described membrane-bound electron transport system in methanogens and of assimilatory type sulfite reductases in certain haloarchaea. Among archaea, only methanogens carried Dsr-LPs. They also possessed homologs of sulfate activation and reduction enzymes. This suggested a shared evolutionary history for methanogenesis and sulfate reduction, and Dsr-LPs could have been the source of the oldest (3.47-Gyr ago) biologically produced sulfide deposit.

## Introduction

Hydrogen-dependent dissimilatory sulfate reduction (4H_2_+SO_4_^2−^+H^+^→HS^−^+4H_2_O) is one of the oldest energy conserving respiratory systems on Earth that developed about 3.5 billion years ago [1], [2], [3], [4]. Sulfite is an obligate intermediate in this process (SO_4_^2−^→ SO_3_^2−^→ HS^−^) and also highly toxic to all types of cells [5]. Therefore, the first organism to develop dissimilatory sulfate reduction ability certainly had invented or acquired the SO_3_^2−^→ HS^−^ conversion system and dissimilatory sulfite reductase gene (*dsr*) in advance. This would also be true for assimilatory sulfate reduction. Accordingly, primary structures of the sulfite reductases have been used to track the evolutionary history of biological sulfate reduction process. It is generally considered that the dissimilatory sulfate reduction system including *dsr* originated in the bacteria, and the sulfate reducing archaea acquired these through horizontal gene transfer [3], [6]. However, the results from a genomic analysis of the methanogenic archaea as reported here puts this concept in doubt.

**Figure 1:**
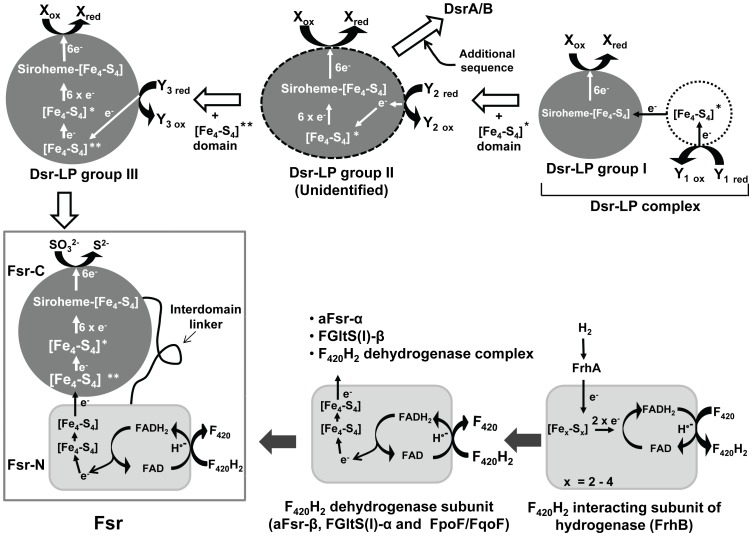
Development of dissimilatory sulfite reductase-like protein (Dsr-LP), F_420_H_2_-dependent sulfite reductase (Fsr) and dissimilatory sulfite reductase (Dsr). Fsr-N and Fsr-C: N-terminal and C-terminal halves of Fsr, respectively. FGltS(I)-α and FGltS(I)-β: F_420_H_2_ dehydrogenase and glutamate synthase subunit of a putative F_420_H_2_-dependent glutamate synthase of methanogens; FpoF/FqoF: F_420_H_2_ dehydrogenase subunit of a membrane-bound proton pumping F_420_H_2_ dehydrogenase complexes of late evolving euryarchaea [36]; aFsr-α and aFsr-β: aSir and F_420_H_2_ dehydrogenase subunits of a putative F_420_H_2_-dependent assimilatory type siroheme sulfite reductase found in haloarchaea. * and ** are peripheral and additional iron sulfur cluster [Fe_4_-S_4_], respectively. Filled and unfilled boxed arrows show the path for the development of Fsr-N and Fsr-C, respectively. Dashed oval or circle, unidentified protein. X and Y_1–3_, unknown electron acceptors and donor, respectively.

Hydrogenotrophic methanogenesis (4H_2_+CO_2_ → CH_4_+2H_2_O) is also one of the oldest energy conserving respiratory systems of Earth developing at least 2.7–3.2 billion years ago [2]. In general, methanogenesis and sulfate reduction are incompatible with each other because sulfite inhibits methanogenesis [7]. On the other hand, the geological data indicate that methanogens of early Earth had to be sulfite tolerant and this ability continues to be important in the deep-sea hydrothermal vent environment that mimics some aspects of early Earth. The development of a fully oxic atmosphere on Earth seemed to have been preceded by a protracted oxygenation period [8], [9], [10] where a small supply of oxygen was quickly and fully sequestered by a high level of sulfide. Such a reaction could lead to incomplete oxidation sulfide and produce sulfite. The vent fluid is rich in nutrients for methanogens, but its temperature, which is 300–350°C [11], is not conducive for the survival of a living cell. However, a mixing of this hot fluid with cold seawater that permeates through the chimney wall provides more hospitable temperatures in some areas within the chimney where hyperthermophilic methanogens grow [12], [13]. The small amount of oxygen brought into the vent by the seawater is neutralized through its reaction with sulfide, which is present in the vent fluid at high levels (5–7 mM) [11]. This reaction helps to maintain anaerobic and low redox potential conditions that are required for the growth of a methanogen, but, as described above, it could generate sulfite. Therefore, one would expect the methanogens of early Earth and extant methanogens of hydrothermal vents to be resistant to sulfite. Indeed certain thermophilic deeply rooted methanogens not only tolerate sulfite but can also use this oxyanion as the sole sulfur source [14], [15], [16]. For *Methanocaldococcus jannaschii*, a hydrogenotrophic and autotrophic methanogen that lives in the deep-sea hydrothermal vents, this ability is due to a new type of sulfite reductase (Fsr) that utilizes coenzyme F_420_ as the electron carrier [15], [17] (Fig. 1). Coenzyme F_420_ is a deazaflavin derivative that is found in every methanogen [18]. At the ground state it functions as a NAD(P) type two-electron (hydride) transfer coenzyme [18]. Fsr homologs are present in sulfite resistant methanogens, and heterologous expression of this enzyme allows a sulfite-sensitive methanogen to tolerate sulfite and to use it as sulfur source [15], [17]. With the discovery of Fsr we inquired how widely the sulfite reduction capabilities or sulfite reductase genes are present in the methanogens and found that ORFs with essential elements of siroheme sulfite reductases are wide spread in this group of euryarchaea. These ORFs show a logical path for the development of a variety of sulfite reductases, including Fsr. These data and additional corroborating evidences suggest an intertwined evolutionary history of methanogenesis and sulfate reduction and make sulfite reductase as a primordial enzyme of the methanogenic archaea. In fact, this conclusion now provides a support to a recent proposal that the first incident of vigorous biological sulfate reduction that occurred at about 2.7 billion years ago was preceded by much earlier occurrence of such an event of minor magnitude [19]. The reported analyses also identified two more putative F_420_-dependent enzymes, one of them being an assimilatory-type sulfite reductase in late evolving archaea.

## Results and Discussion

The synthesis presented below is based on the known structure-function relationships of Fsr, dissimilatory sulfite reductases and assimilatory sulfite reductases (Dsr and aSir) [20], [21], [22], [23]. The terms of Dsr and aSir traditionally refer to the determined physiological roles as well as distinct structural types [20]. However, in some cases, such as in Fsr, Dsr type structures have been found to be associated with assimilatory functions. In this report the terms Dsr and aSir refer to the structural features and not necessarily the physiological functions.

### Search for the origin of Fsr

#### Distribution of Fsr homologs in methanogens

**Figure 2:**
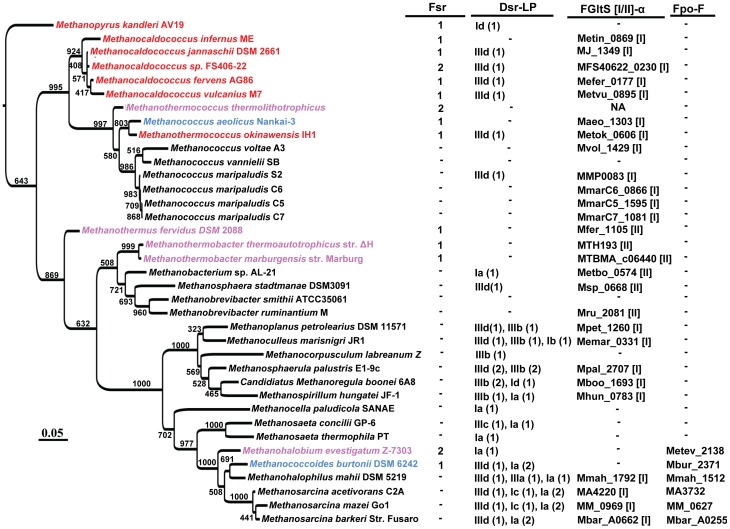
Distribution of Dsr-LP, Fsr, FGltS(I/II)-α and FpoF in methanogenic archaea. The information has been presented on a 16S rRNA sequence based phylogenetic tree of methanogens for which whole genome sequences are available. *Desulfurococcus fermentans* was used as an outgroup. The confidence values presented at the branches of the tree were estimated from 1000 bootstrap repetitions; the scale bar underneath the tree indicates the number of base substitutions per site. (1 or 2), number of each type of sulfite reductase homolog in a methanogen. Dsr-LP: dissimilatory sulfite reductase-like proteins; Fsr, FGltS(I/II)-α, and FpoF: same as in the legend of Fig. 1. The Dsr-LP group numbers (Ia-d and IIIa-d) are according to Fig. 3. Color representation of Fsr-containing methanogens (color, characteristic); red, hyperthermophilic vent methanogen (except *M. okinawensis* is a thermophile); lavender, thermophile; blue, mesophile and psycrophile. Classification of FGltS according to Fig. 6 is shown in square brackets.

The Fsr homologs had a significant presence within the methanogenic archaea and they could be considered a specialty of hyperthermophilic methanogens from the deep-sea hydrothermal vents and certain halophilic methanogens (Fig. 2). Only exceptions are *Methanothermobacter thermoautotrophicus* and *Methanothermus fervidus* which are freshwater thermophiles from a municipal sewage digester and a hot spring, respectively [24], [25]. However, *M. thermoautotrophicus* can grow at high salt concentrations [26]. *M. fervidus* has not been tested for halotolerance. Every deep-sea hydrothermal vent methanogen, including every *Methanocaldococcus* species, carried at least one Fsr homolog (Fig. 2); *Methanothermococcus thermolithotrophicus* [27] and *Methanohalobium evestigatum* Z-7303 [28], which are moderate thermophiles isolated from geothermally heated sea sediments, and salt lagoon respectively, and *Methanocaldococcus sp.* FS406-22 [29], a hydrothermal vent hyperthermophile, encode two Fsr homologs (Fig. 2). One of the two Fsr homologs of *M. thermolithotrophicus* is likely to be a nitrite reductase, because the organism can use nitrate as nitrogen source [30], a sulfite reductase often can reduce nitrite [20], and Fsr from *M. jannaschii* has been found to reduce nitrite with F_420_H_2_ (Eric F. Johnson and Biswarup Mukhopadhyay, unpublished observation).

Three non-hydrothermal vent methanogens, *Methanohalobium evestigatum* (moderate thermophile) [31], *Methanococcus aeolicus* Nankai-3 (mesophile) [32], and *Methanococcoides burtonii* DSM 6242 (psychrophile) [33], that carried Fsr homologs (Fig. 2) live in marine environments. It is likely that they have acquired *fsr* via horizontal gene transfer from the vent methanogens. Cooler seawater is known to disperse vent organisms from one vent field to another and the strictly anaerobic vent methanogens can survive oxygen exposure at low temperatures [34]. Therefore, vent methanogens could reach the habitats of mesophilic and psychrophilic marine methanogens. However, the genomic sequences at the immediate vicinity of *fsr* do not show conservation. This could indicate that the above-mentioned non-hydrothermal vent methanogens received *fsr* from vent methanogens early in their development or both groups arose from a common ancestor that carried *fsr* and continued genome evolution removed the context similarity. An Fsr homolog (GZ27A8_52) is present in ANME-1 [15], an uncultured archaeon that is a component of a consortium that performs anaerobic oxidation of methane [35]. This observation raises the possibility of sulfite and even sulfate reduction coupled anaerobic oxidation of methane in this halophile from permanently cold methane rich marine sediment. This possibility is supported by the observation that *Methanococcoides burtonii*, a methanogen that is phylogenetically closely related to ANME-1 and lives in an environment that is similar to the habitat of ANME-1 [33], also carries an Fsr homolog (Fig. 2).

#### A chimeric structure of Fsr likely developed through gene fusion

The N-terminal half of *M. jannaschii* Fsr (Fsr-N; residues 1–311 of ORF MJ_0870) is a homolog of a free-standing polypeptide called F_420_H_2_ dehydrogenase (FpoF/FqoF) that is found in late evolving methylotrophic and acetotrophic methanogens and *Archaeoglobus fulgidus*, a sulfate reducing archaeon closely related to the methanogens [15], [17], [36] (Fig. 1). In these organisms FpoF/FqoF serves as the electron input subunit of a membrane-bound NADH-dehydrogenase type energy transduction system called F_420_H_2_ dehydrogenase complex [15], [17], [36]. The C-terminal half of Fsr (Fsr-C; residues 325–620 of MJ_0870) is a homolog of Dsr [15], [17] (Figs. 1 and S1). Fsr functions both as a dissimilatory (detoxification) and an assimilatory (sulfide nutrition) enzyme [15], [17] and Fsr-C does not show significant sequence similarity to aSir [15]. Two documented partial reactions of Fsr, namely dehydrogenation of F_420_H_2_ and reduction of sulfite [15] suggest that Fsr-N retrieves electrons from F_420_H_2_ and reduces FAD, and then via the Fe-S centers of Fsr-N and Fsr-C, the electrons from FADH_2_ are transferred to siroheme of Fsr-C where sulfite is reduced to sulfide; FAD acts as 2-electron/1-electron switch connecting 2-electron-donating F_420_H_2_ and 1-electron-carrying Fe-S centers. Functionally Fsr reaction mimics NADH-dependent reduction of sulfite by *E. coli* aSir which is composed a siroheme-containing protein subunit (Sir-HP) and a flavoprotein subunit (Sir-FP) [20]; Sir-HP and Sir-FP are equivalent to Fsr-C and Fsr-N, respectively. However, as mentioned above Fsr-C and Sir-HP do not share a significant sequence homology, and Fsr-N and Sir-FP are also not homologous to each other [15].

#### Strategy for a search for the origin of Fsr

The chimeric nature of Fsr and the logic that simpler units would arrive first suggested that Fsr was built from pre-existing parts, Fsr-N and Fsr-C. These parts were either available in the methanogens or were transferred horizontally to these archaea from other organisms. The latter possibility seemed weak because Fsr homologs had a significant distribution within the methanogenic archaea and apparently absent in the eukaryotic and bacterial domain as well as in other members of the archaea. However, for Fsr to be a true invention of the methanogens, Fsr-N and Fsr-C homologs must be at least widespread if not fully restricted to these organisms. The results presented below show that this is indeed the case.

### Search for Fsr-C homologs: discovery of Dsr-LP, a family of dissimilatory sulfite reductase like ORFs in methanogens

**Figure 3:**
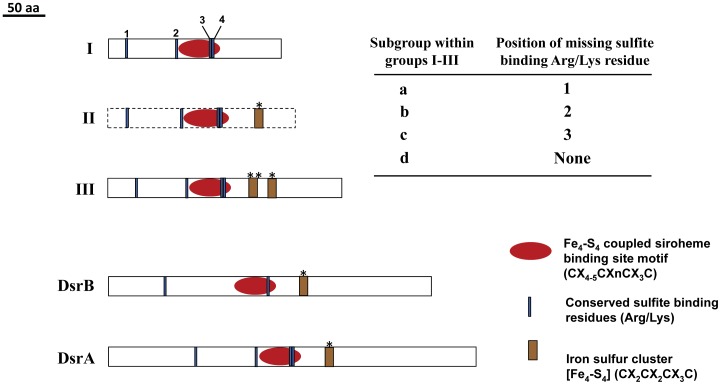
Groups of Dsr-LP and Dsr. The Dsr-LP classification (groups Ia-d and IIIa-d) is based on the presence and absence of the following functionally important sequence signatures: [Fe_4_-S_4_]-coupled siroheme binding site; iron sulfur cluster sites (*, peripheral; ** additional); and sulfite binding amino acid residues (Arg or Lys). Numbers 1–4 indicates the positions (1^st^, 2^nd^, 3^rd^ and 4^th^) of sulfite binding amino acid residues. The amino acid sequences representing these characteristics are shown in Fig. S1. Groups IIa-d, represented by dotted-line box are hypothetical and yet to be detected.

The homologs of Fsr-C as freestanding units were abundant in the methanogens and they were diverse in their contents of the characteristic sequence features (Figs. 3 and S1). About 67% of the fully closed methanogen genomes examined carried these homologs and their total number was 49 (Fig. 2). Since their catalytic and *in vivo* functions are not known, these ORFs with high sequence similarities with Dsr subunits (DsrA and DsrB) and low similarities with aSir were named Dsr-LP (dissimilatory sulfite reductase-like proteins). The Dsr-LP ORFs were compared with the DsrA/B sequences to locate their relevant structural features (Figs. 3 and S1). Dsr-LPs as a family were found to carry all of the following defining structural features of sulfite reductases: i. A coupled siroheme-iron sulfur cluster where sulfite or nitrite is reduced; ii. An iron-sulfur cluster (called peripheral [Fe_4_-S_4_] cluster) that shuttles electrons from a donor to the oxyanion reduction site; and iii. Arg/Lys residues that facilitate the binding of negatively charged sulfite [20], [21], [22], [23], [37]. Fig. S1 shows the amino acid residues and sequence motifs representing these features in well-studied Dsr and their parallels in Dsr-LP and Fsr-C. In this comparison the Dsr-LPs fell into two broadly defined groups I and III (Fig. 3). As discussed below, the development of group III from group I had likely proceeded through an intermediate state and to represent this state we have proposed group II Dsr-LP (Figs. 1 and 3). We have not found a representative for group II thus far. Every Dsr-LP carried the sequence motif for the coupled siroheme-iron sulfur cluster, the most defining feature of sulfite reductases. Group I had the simplest features as the members carried conserved signatures for siroheme-iron sulfur cluster but lacked ferredoxin domains (Figs. 3 and S1). The hypothesized group II would have the group I features and the peripheral [Fe_4_-S_4_] cluster (see * in Figs. 3 and S1). Group III was characterized by a siroheme-iron sulfur cluster, a peripheral Fe_4_-S_4_ cluster and an additional [Fe_4_-S_4_] cluster (Figs. 3 and S1). The members of each group carried either three or all of the four Arg/Lys residues that define sulfite-binding sites in Dsr [21], [22], [23], [37] and based on the positions of the missing Arg/Lys residue (1^st^–4^th^, counting from the NH_2_-terminus of the polypeptide; Figs. 3 and S1) they were further classified in four sub-groups. Sub-groups a, b, and c lacked the 1^st^, 2^nd^, and 3^rd^ sulfite-binding Arg/Lys residue, respectively, and d, carried all four residues (Fig. 3). The 4^th^ sulfite-binding residue was fully conserved across all types of sulfite reductases and therefore could be critical to the binding of an anionic substrate, sulfite or nitrite. Group IIId could be considered the precursor of Fsr-C. DsrA/B lack the additional iron-sulfur cluster (See ** in Figs. 3 and S1) and the significance of the presence of this unit in groups III Dsr-LP is discussed below.

**Figure 4:**
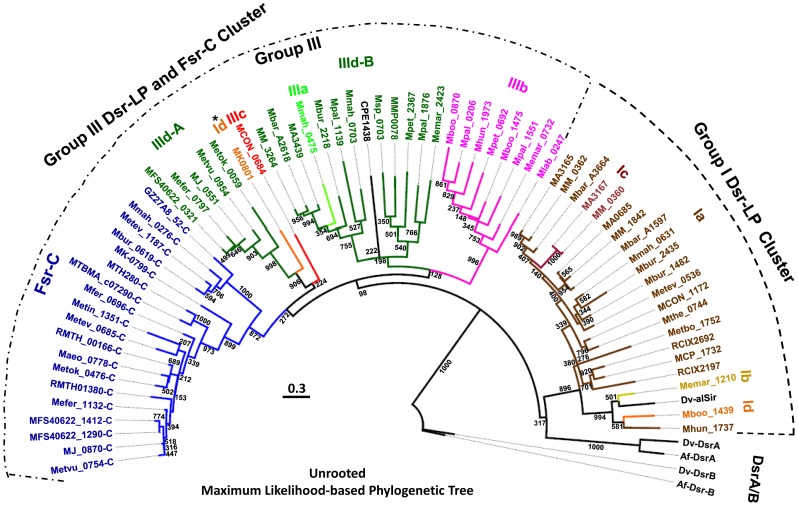
Phylogenetic tree of Dsr-LP and Dsr based on Maximum Likelihood method. Dsr-LP (Groups Ia-d and IIIa-d) and Dsr, defined in Fig. 3 and its legend. Dsr and Fsr-C, defined in the legend of Fig. 1. Dv-DsrA/B and Af-DsrA/B, Dsr subunits A and B of *Desulfovibrio vulgaris* strain Hildenborough (ORFs DVU0402 and DVU0403) and *Archaeoglobus fulgidus* DSM 4304 (ORFs AF0423 and AF0424), respectively [64], [65]; Dv-alSir and CPE1438, anaerobic small sulfite reductase of *Desulfovibrio vulgaris* strain Hildenborough (ORF DVU_1597) and *Clostridium perfringens* strain 13, respectively [66]; The ORF numbers followed by “-C”, Fsr-C homologs. Abbreviation for organism names preceding the listed ORF numbers: MTBMA, *Methanothermobacter marburgensis* strain Marburg; MTH, *Methanothermobacter thermautotrophicus* ΔH; Metbo, *Mehanobacterium* sp. AL-21; RMTH, *Methanothermococcus thermolithotrophicus* (sequence obtained from Dr. William B. Whitman, University of Georgia); Mfer, *Methanothermus fervidus* DSM 2088; Maeo, *Methanococcus aeolicu*s Nankai-3; Msp, *Methanosphaera stadtmanae* DSM 3091; MMP, *Methanococcus maripaludis* S2; Mpal, *Methanosphaerula palustris* E1-9c*;* Mpet, *Methanoplanus petrolearius* DSM 11571*;* Memar, *Methanoculleus marisnigri* JR1; MM, *Methanosarcina mazei* Gö1; MA, *Methanosarcina acetivorans* C2A; Mbar, *Methanosarcina barkeri* strain Fusaro*;* Mbur, *Methanococcoides burtonii* DSM 6242; Mmah, *Methanohalophilus mahii* DSM 5219; Metev, *Methanohalobium evestigatum* Z-7303; Mthe, *Methanosaeta thermophila* PT; MCON, *Methanosaeta concilii* GP-6; MCP, *Methanocella paludicola* SANAE; Mboo, *Candidiatus Methanoregula boonei* 6A8; Mlab, *Methanocorpusculum labreanum* Z; Mhun, *Methanospirillum hungatei* JF-1; Metbo, *Methanobacterium* sp. Al-21; Metok, *Methanothermococcus okinawensis* IH1; Metin, *Methanocaldococcus infernus* ME; MFS40622, *Methanocaldococcus sp.* FS406-22; MJ, *Methanocaldococcus jannaschii* DSM 2661; Mefer, *Methanocaldococcus fervens* AG86; Metvu, *Methanocaldococcus vulcanius* M7; MK, *Methanopyrus kandleri* AV19; GZ27A8_52, uncultured archaeon related to *Methanosarcina* and a member of an anaerobic methane oxidizing consortium; RCIX2692 and RCIX2197, uncultured methanogenic archaeon RC-1 and primary methane producer in rice rhizosphere. The bootstrap value shown at each branch is from 1000 replicates. Scale bar, number of amino acid substitutions per site. *, shows outliers. A and B, sub-clades of group IIId.

**Figure 5:**
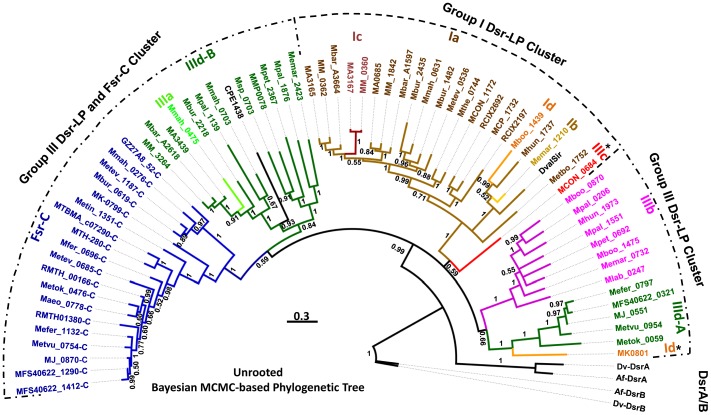
Phylogenetic tree of Dsr-LP homologs based on Bayesian Markov chain Monte Carlo (MCMC) analysis. ORF numbers and all abbreviations used are described in the legend of Fig. 4. For each branch a posterior probability value (0–1) is shown. Scale bar, number of amino acid substitutions per site.

To gain better understanding on the evolutionary relationships between Dsr-LPs and Fsr-C homologs, we have performed phylogenetic analysis using two different approaches, namely Maximum likelihood (ML) (Fig. 4) and Bayesian Markov chain Monte Carlo (MCMC) phylogenetic inference (Fig. 5). Results from both approaches presented group I Dsr-LPs and Fsr-C as monophyletic (Figs. 4 and 5). Phylogenetically, group III was composed of more heterogeneous members, although individual sub-groups formed tighter clades. These observations allude to the following possibilities: i. Group I members are under strong selective pressures; ii. Group III members evolved from group I to provide functional diversity and the path of their development will be apparent when members of group II, the missing links, are identified.

Both ML and MCMC analyses divided group IIId into two sub-clades, A and B (Figs. 4 and 5), where IIId-A represented almost exclusively the hyperthermophilic methanogens from deep-sea hydrothermal vents and the member of the sub-clade IIId-B were mostly from evolutionarily late evolving mesophilic methanogens. ML tree presented group IIId-A as closely related to Fsr-C, whereas in MCMC tree group IIId-B was linked to Fsr-C. However, both Fsr and IIId-A exist in hyperthermophilic methanogens, and therefore, the relationship presented by ML analysis is more reliable.

Among the structural sub-groups a-d (Figs. 4 and 5) only Ia, IIIb, and IIId were well represented and formed tight clades. Members of other sub-groups were rare and they showed a diversity of phylogenetic positions. In both ML and MCMC analysis MK0801 of *M. kandleri*, the sole member of group Id, was grouped with group IIId-A. Often *M. kandleri*, the most thermophilic methanogen (maximum growth temperature, 110°C) and an inhabitant of deep-sea hydrothermal vents, is considered the most deeply-rooted methanogen [38], [39], [40], [41]. It is possible that MK0801 and group IIId-A Dsr-LPs arose from a common ancestor.

### Distribution of Dsr-LP in the methanogens

The distribution of Dsr-LP in the methanogens exhibited a distinct pattern (Fig. 2). The deeply rooted organisms belonging to the classes of Methanopyri (*Methanopyrus kandleri*) and Methanococci (genera of *Methanocaldococcus*, *Methanotorris*, *Methanothermococcus*, and *Methanococcus*) carried limited numbers of sulfite reductase homologs. Each of the *Methanocaldococcus* species carried at least one Fsr homolog and a Dsr-LP, except *Methanocaldococcus infernus* lacked Dsr-LP. *Methanopyrus kandleri* possessed one Fsr and one Dsr-LP. With the exception of *Methanococcus maripaludis* strain S2, all *Methanococcus* species lacked Dsr-LP, and Fsr was absent in this genus. Of the two methanothermococci studied thus far, one carried two Fsr and lacked Dsr-LP and the other had one homolog for each of Fsr and Dsr-LP (Fig. 2); these scenario might change as these two genomes have not yet been fully sequenced. Within the Methanobacteria class, each of *Methanothermobacter* and *Methanothermus* species carried an Fsr homolog and lacked Dsr-LP homolog. *Methanobrevibacter* genomes were devoid of sulfite reductase homologs (Fsr or Dsr-LP) whereas *Methanosphaera stadtmanae* carried one Dsr-LP and was devoid of Fsr. The Dsr-LP was highly prevalent in the class of Methanomicrobia, each member carrying 1–4 homologs of this protein; Fsr was only rarely found in this group. The genomes of *Methanosarcina and Methanosphaerula* species encoded the maximum number Dsr-LP proteins (four homologs) and lacked Fsr. Methanogens that contained more than two Dsr-LP homologs are mostly mesophilic and psycrophilic. It seems that in these late evolving organisms Dsr-LP underwent recruitment to multiple needs.

### Fsr-N homologs: widespread in methanogens and being parts of two additional and novel enzymes, putative F_420_-dependent glutamate synthase (FGltS) and assimilatory type Fsr (aFsr)

**Figure 6:**
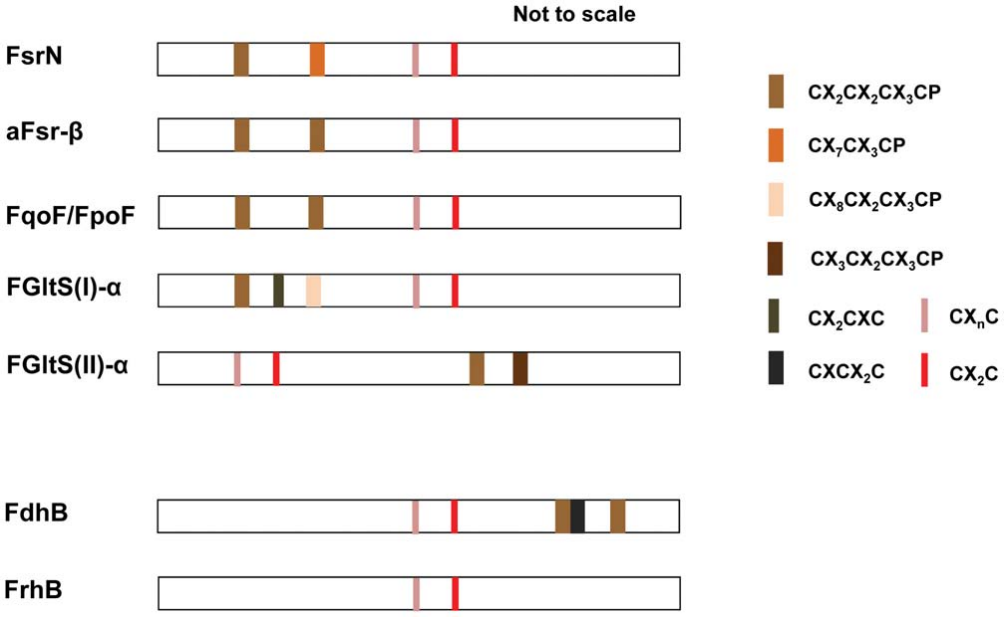
Groups of Fsr-N homologs. The sketches are based on respective amino acid sequence characteristics shown in Fig. S4. FGltS(I)-α and aFsr-β: F_420_H_2_-dehydrogenase subunit of a putative F_420_H_2_-dependent glutamate synthase of methanogens and a putative F_420_H_2_-dependent assimilatory type siroheme sulfite reductase found in haloarchaea; FpoF/FqoF: F_420_H_2_ dehydrogenase subunit of a membrane-bound proton pumping F_420_H_2_ dehydrogenase complex of late evolving euryarchaea [36]. Note: FGltS(II)-α departs significantly from Fsr-N in primary sequence and it is not included in Fig. S4. F_420_-interacting or β subunits of F_420_H_2_ reducing hydrogenase (FrhB) and formate dehydrogenase (FdhB) [67], [68] are shown for comparison.

The identification of homologs of Fsr-N via automated similarity (BLAST) searches proved difficult because it is highly similar to the F_420_-interacting subunits of F_420_-dependent hydrogenases (FrhB) and formate dehydrogenases (FdhB) [15]. In fact this factor has led to the misconception that FpoF/FqoF, the closest relative of Fsr-N [15], is absent in the strict hydrogenotrophic methanogens belonging to the orders of Methanobacteriales and Methanococcales and is a specialty of the methylotrophic methanogens of the Methanomicrobia class [42]. We circumvented this problem by using the following primary sequence relationships: Fsr-N = FrhB+two additional ferredoxin-type [Fe_4_-S_4_] centers at the N-terminus; FdhB = FrhB+two additional [Fe_4_-S_4_] centers at the C-terminus (Fig. 6). This analysis showed that in the methanogens freestanding Fsr-N homologs were as abundant as Dsr-LPs (Figs. 2, S2 and S3).

Based on both Maximum Likelihood and Bayesian Markov chain Monte Carlo (MCMC) analyses, phylogenetically Fsr-N homologs fell into four distinct clades (FGltS(I)-α – aFsr-β – Fpo/FqoF; Fsr-N; FGltS(II)-α – FrhB; and FdhB) (Figs. S2 and S3), each of which was linked to a specific genomic context. From these clues the following two new putative F_420_-dependent enzymes were located: an F_420_-dependent glutamate synthase (FGltS(I)) in methanogens (L-glutamine+α-ketoglutarate+F_420_H_2_ → 2 L-glutamate+F_420_), where FGltS(I)-α is the Frs-N homolog and FGltS(I)-β is the glutamate synthase subunit; an assimilatory Fsr (aFsr) of the halobacteria where the sulfite reductase subunit (aFsr-α) is aSir type and the electron retrieving subunit (aFsr-β) is a Fsr-N homolog. This is the first report for aFsr and FGltS(I); previously characterized glutamate synthases use NADH, NADPH or reduced ferredoxin as the reductant [43]. Methanogen genomes encode another version of putative F_420_-dependent glutamate synthase where F_420_-interacting subunit is an FdhB homolog (Fig. 6) and here it has been named FGltS(II); a homolog of FGltS(II) has recently been found in *Methanothermobacter marburgensis* genome [44].

As shown in Figs. 6 and S4, among the Fsr-N homologs FrhB has the simplest structure. It also exists in every methanogen. Accordingly, we propose that from FrhB more complex Fsr-N homologs evolved through recruitment of additional iron-sulfur centers, and therefore, FrhB is the ancestor of all Fsr-N homologs (Fig. 1).

### The additional ferredoxin domain of group III Dsr-LPs: evolution of an inter-domain electron transfer conduit of Fsr

Group III Dsr-LPs showed the potential of assembling an additional [Fe_4_-S_4_] element (shown by ** in Figs. 1, 3 and S1) which has not been found in archaeal and bacterial DsrA and DsrB subunits. For Fsr-C this additional [Fe_4_-S_4_] cluster likely allows electron transfer between the Fsr-N and Fsr-C domains via the following path (Fig. 1): F_420_H_2_ → FAD (in Fsr-N) → *additional [Fe_4_-S_4_]* → peripheral [Fe_4_-S_4_] cluster → siroheme-coupled [Fe_4_-S_4_] → siroheme → sulfite. All of these steps except the first two operate in Dsr, which obtains electrons from iron-sulfur proteins [21], [22], [23].

### Development of Fsr and aFsr

Based on the findings reported above, it is likely that Fsr is an invention of the methanogens and it was built in a methanogen from a group IIId Dsr-LP and a Fsr-N homolog that existed in these archaea (Fig. 1); above described inter-domain electron transfer conduit was a key for this development. It is possible that the process involved an intermediate complex composed of Fsr-N and Fsr-C as individual subunits followed by a gene fusion event that generated the complete Fsr polypeptide; the discovery of a putative aFsr composed of Fsr-N and aSir subunits in the halobacteria provides further support to this hypothesis. Similar to Fsr, aFsr is also likely to be an archaeal invention, as an aSir homolog that is found in *Sulfolbus solfataricus*, a crenarchaeon, appears at the most basal position in a phylogenetic tree that covers archaeal, bacterial and eukaryotic aSirs [45].

### Evolution of sulfite reductases: methanogens being the likely host for this development

The Dsr-LP ORFs were widely present in the methanogens (Fig. 2) and they represented from the very minimum to the most complex forms of Dsr proteins encountered in extant archaea and bacteria (Figs. 1, 3, and S1). Every functional unit of sulfite reductases, especially the Dsr, is represented by the Dsr-LPs (Figs. 3 and S1). Therefore, sulfite reductase type proteins have a long evolutionary history in methanogens and most likely one such ORF was present in their common ancestor or was acquired by this organism at a very early stage of evolution. This is consistent with the observation that the crenarchaeal DsrA and DsrB from *Pyrobaculum* species occupy the most basal position in a phylogenetic tree for archaeal and bacterial Dsr subunits [45]. The group I Dsr-LPs with the simplest attributes (coupled siroheme-iron sulfur cluster and sulfite binding Arg/Lys residues) could be considered as the recognizable earliest forms of sulfite reductases (Figs. 1, 3, and S1). Then through association and subsequent fusion with a ferredoxin (Fe_4_-S_4_) unit these forms gave rise to group II Dsr-LPs (Fig. 3), which remains unidentified (Figs. 1 and 3). Insertion of an additional ferredoxin (Fe_4_-S_4_) unit into group II Dsr-LPs resulted into III Dsr-LPs. Similarly, an expansion of the primary structure converted group II Dsr-LPs to DsrA/B (Figs. 1 and 3).

In a recent effort to locate the most ancestral sulfite reductase in the extant organisms the amino acid sequences at and around the coupled siroheme-iron sulfur clusters of several of these proteins were phylogenetiocally analyzed [45]. In this analysis the small size monomeric assimilatory sulfite reductase of *Desulfovibrio vulgaris* (Dv-alSir) that carries a coupled [Fe_4_-S_4_]-siroheme center but lacks the peripheral [Fe_4_-S_4_] center was found to be the most deeply rooted in the aSir clade [45] and accordingly this simpler protein was considered as the recognizable earliest ancestor for all types of sulfite reductases. The anaerobic sulfite reductase (AsrC) which is expressed from the *asrABC* operon in *Salmonella typhimurium* and several *Clostridium* species was placed in a distinct clade. In our analysis we took a different approach. Since coupled [Fe_4_-S_4_]-siroheme center of sulfite reductases has not been altered significantly through about 3.5 billion years [19], [45], we considered full amino acid sequences of sulfite reductase proteins to obtain the clues to the evolutionary processes that have shaped the sulfite reductases of the methanogens and other organisms. In this analysis, alSir (DVU_1597 or Dv-alSir of *Desulfovibrio vulgaris*), AsrC (CPE1438 of *Clostridium perfringens*), Dsr-LP and Fsr-C were found to have Dsr type structural features (Figs. 3 and S1). Dv-alSir was similar to group I Dsr-LP, our proposed ancestral form, and AsrC was a group III Dsr-LP, possessing the more complex features. These observations are consistent with the previous report [45]. In the bacterial domain Dsr-LP homologs were found only in anaerobic or facultative anaerobes belonging to the phyla of Firmicutes (such as *Clostridium*, *Desulfitobacterium*, *Desulfotomaculum*, and *Veillonella*) and Proteobacteria (such as *Geobacter*, *Desulfovibrio*, and *Syntrophus*). Most of the bacterial Dsr-LP homologs were AsrC or group III Dsr-LP type. All these bacteria are likely to associate with the methanogens in nature. In this context we note that within the archaeal domain Dsr-LPs are almost fully restricted to the methanogens (Phylum, Euryarchaeota) where they are widespread. Therefore, it is likely that the simplest form of sulfite reductase was generated in a methanogen or was acquired by these archaea from anaerobic bacteria early in their evolution. The strict and apparently long association of Dsr-LPs with methanogens alludes to certain functions that are ecologically important to these organisms.

*M. maripaludis* S2 does not carry an Fsr but possesses a group IIId Dsr-LP (ORF MMP0078) and it is sensitive to sulfite. When *M. jannaschii* Fsr is expressed in *M. maripaludis*, the recombinant tolerates sulfite and even uses this oxyanion as sulfur source [17]. Hence, *M. maripaludis* not only folds Fsr properly but also assembles siroheme in the recombinant protein. Hence, it synthesizes a siroheme of the type that is recognized by the Dsr-LP domain of Fsr. It is likely that in wild-type *M. maripaludis* this cofactor is assembled in MMP0078, the only potential siroheme protein in this organism. By inference the same property could be expected for all Dsr-LP carrying methanogens (Fig. 2). Therefore, not only the sulfite reductase, but also siroheme, the most crucial part of this enzyme, is an ancient component of the methanogens.

Most methanogens carry the homologs of two key enzymes for the reduction of sulfate to sulfite, sulfate adenylyltransferase (Sat) and adenosine 5′-phosphosulfate (APS) reductase (Apr) [15]. The Apr homolog of *M. jannaschii* (MJ_0973) exhibits the relevant activity, albeit with a low V_max_ and k_cat_ values [46]. Therefore, it is likely that the intertwined history of methanogens with sulfite reductase extends up to the full-scale sulfate reduction pathway. This hypothesis is consistent with a recent proposal about the first incident of sulfate reduction on Earth [19]. Analysis of isotope records of sedimentary sulfides had identified a major microbial sulfate reduction event starting by 2.7 Gyr ago [47]. However, 3.47-Gyr old barites from North Pole, Australia, have been found to carry biologically produced sulfide which has been taken as an indication of a more ancient but minor sulfate reduction process [19]. From our results it could be hypothesized that this signature for ancient biogenic sulfide originated from minor sulfate reduction activities of the above-mentioned machineries of methanogens. Sulfite reduction had to develop before sulfate reduction for avoiding sulfite toxicity. Therefore, it is also possible that the ancient sulfide originated from reversible conversion of sulfite to sulfide catalyzed by an ancient Dsr-LP that led to an isotope signature (via fractionation [48]). If one or both of these hypotheses were proven to be true, the relationships of two of the most ancient respiratory metabolisms of earth, hydrogenotrophic methanogenesis and sulfate reduction, would be at least 3.47 Gyr old. Dsr-LPs have the essential features of Dsr and yet even their most complete versions do not enable methanogens to reduce sulfite [17]. A Dsr-LP type protein has been purified from *Methanosarcina barkeri* and it shows weak sulfite reductase activity with reduced methylviologen, an artificial electron carrier, as reductant [49]. However, the physiological relevance of this observation is unclear as *M. barkeri* is sensitive to sulfite and cannot use this oxyanion as sulfur source [17]. Since the synthesis of coenzyme M requires sulfite [50], it is possible that Dsr-LP is used to meet this need via the oxidation of sulfide. An example of the oxidation of sulfide to sulfite via a membrane-bound siroheme sulfite reductase complex has been found in *Allochromatium vinosum*, a phototrophic sulfur bacterium [51], [52], [53], [54], [55], [56], [57]. However, certain methanogens do not carry either Dsr-LP or Fsr (Fig. 2) and this fact questions the role of Dsr-LP in the synthesis of coenzyme M which is essential for methanogenesis, the only source of energy for methanogens [58]. It is conceivable that Dsr-LPs carry out certain heme-driven electron transport functions that relate someway to sulfite reduction.

## Methods

Multiple sequence alignment was performed using MUSCLE at the European Bioinformatics Institute web server [59]. Regions with poor alignments were removed using Gblocks0.91b [60]. Amino acid sequence for homologs of Mj-Fsr (MJ_0870), Dsr-LP and N-terminal half of Fsr (Fsr-N) were collected via Blastp and Psi-Blast searches into the non-redundant protein database of the National Center for Biotechnology Information (NCBI); MJ_0870 (amino acid residue 1–620 amino acids), C-terminal half of Mj-Fsr (amino acid residue 325–650), and N-terminal half of Mj-Fsr (amino acid residue 1–311), respectively were used as queries. Searches for the homologs of Dsr-LP in bacteria were performed by the use of Psi-Blast with MMP0078 and MK0801 as queries for group I and group III Dsr-LP, respectively.

Phylogenetic trees for Dsr-LPs, Fsr-N homologs, and 16S rRNAs of methanogens were constructed by Maximum Likelihood method using Phylip 3.69 [61] employing Proml and Dnaml with default parameters, respectively. The 16S ribosomal RNA of *Desulfurococcus fermentans*, a crenarchaeon [62], was used as an outgroup for building the 16S rRNA tree. Bootstrap values were estimated by Seqboot with 1000 replicates. Consensus trees were generated by Consense. Figtree v1.3.1, downloaded from http://tree.bio.ed.uk/software/Figtree, was used to view the phylogenetic trees.

For additional phylogenetic analysis, we employed Bayesian Markov chain Monte Carlo (MCMC) method that was implemented with MrBayes 3.2 [63]. In brief, the WAG model, the best-fitting amino acid model priors with the highest posterior probability (1.0) was chosen for the amino acid replacement model in our analysis. Searching for the best amino acid model was performed by conducting preliminary runs on MrBayes 3.2 using the option of mixed amino acid model priors. Dsr-LP and Fsr-N sequence datasets were modeled with an independent gamma distribution of substitution rates and were simulated for 2,000,000 and 3,500,000 generations resulting in the “average standard deviation of split frequencies” (ASDSF) of 0.0055 and 0.0085, respectively. ASDSF was used for convergence assessment. A consensus tree was generated from two independent runs using a recommended “burn-in” value of 25%.

## Supporting Information

Figure S1 Primary structure comparison of Dsr-LPs of methanogenic archaea and archaeal and bacterial Dsr. Fsr-C, defined in Fig. 1. Structural type of Dsr-LP groups, as described in Fig. 3, shown as number Ia-d and IIIa-d left to the alignment (groups IIa-d, yet to be detected); “+”, sulfite binding Arg or Lys residues; arrows, residues involved in assembling [Fe_4_-S_4_]-coupled siroheme; over-line, sequence motif involved in assembling [Fe_4_-S_4_] cluster; * and **, peripheral and additional [Fe_4_-S_4_] centers, respectively. Black bullets, conserved cysteine residues for [Fe_4_-S_4_] and siroheme sites. Red bullet, non-conserved cysteine residues coupling [Fe_4_-S_4_] center with siroheme in Dv-DsrB. The details of the abbreviations for organism names are in the legend of Fig. 4. The following color shadings have been used to represent conserved residues: teal, arginine or lysine; dark blue, cysteine; blue, prolin; grey, other residues. The color shadings in the left panel representing various sulfite reductases correspond to the same in Fig. 4.
(TIF)

Figure S2 Phylogenetic tree of homologs of *M. jannaschii* Fsr-N according to Maximum Likelihood method. aFsr-β, FqoF, FpoF, and FGltS(I)-α are Fsr-N homologs. FrhB and FdhB are shown for comparison. See the legend of Fig. 1 for the full names of Fsr-N, aFsrβ, FqoF, FpoF and FGltS-α, and Fig. 6 for FrhB and FdhB. The ORF numbers followed by “-N”, Fsr-N homologs. Abbreviations for organism names preceding the ORF numbers (in addition to those described in Fig. 4 legend): Hbor, *Halogeometricum borinquense* DSM 11551*;* Huta, *Halorhabdus utahensis* DSM 12940; HacJB3, *Halalkalicoccus jeotgali* B3; Htur, *Haloterrigena turkmenica* DSM 5511*;* Rxyl_0964, *Rubrobacter xylanophilus* DSM 9941; Tter, *Thermobaculum terrenum* ATCC BAA-798; GZ27A8_52, uncultured archaeon related to *Methanosarcina* species; MmarC6, *Methanococcus maripaludis* C6; MmarC5, *Methanococcus maripaludis* C5; MmarC7, *Methanococcus maripaludis* C7; Mevan, *Methanococcus vannielii* SB; Mvol, *Methanococcus voltae* A3*;* AF, *Archaeoglobus fulgidus* DSM 4304, Msm, *Methanobrevibacter smithii* ATCC 35061*;* P06130, accession number for *Methanobacterium formicicum* FdhB. The bootstrap value shown at each branch is from 1000 replicates. Scale bar, number of amino acid substitutions per site.
(TIF)

Figure S3 Phylogenetic tree of Fsr-N homologs based on Bayesian Markov chain Monte Carlo (MCMC) analysis. ORF numbers and all abbreviations used are described in the legend of Fig. S2. For each branch a posterior probability value (0–1) is shown. Scale bar, number of amino acid substitutions per site.
(TIF)

Figure S4 Primary structure comparison of homologs of *M. jannaschii* Fsr-N. aFsr-β, FqoF, FpoF and FGltS(I)-α are Fsr-N homologs. FGltS(II)-α, FrhB and FdhB are shown for comparison. The details of the abbreviation for ORF numbers are in the legend of Figs. 4 and S2. The color shadings and colored letters represent conserved and partially conserved amino acid residues, respectively: dark blue, cysteine; turquoise, prolin; red, aspartate and glutamate; green, glycine; orange, phenylalanine or tyrosine; grey, valine or isoleucine. Black bullets, conserved cysteine residues for [Fe_4_-S_4_] sites; over-line, sequence motif involved in assembling [Fe_4_-S_4_] cluster. The color shadings in the left panel representing various proteins correspond to the same in Figs. S2 and S3.
(TIF)
