# Supplementary figures and images for: An Intertwined Evolutionary History of Methanogenic Archaea and Sulfate Reduction
Source: PLoS One. 2012 Sep 21;7(9):e45313. doi: 10.1371/journal.pone.0045313 (PMC3448663; doi:10.1371/journal.pone.0045313)

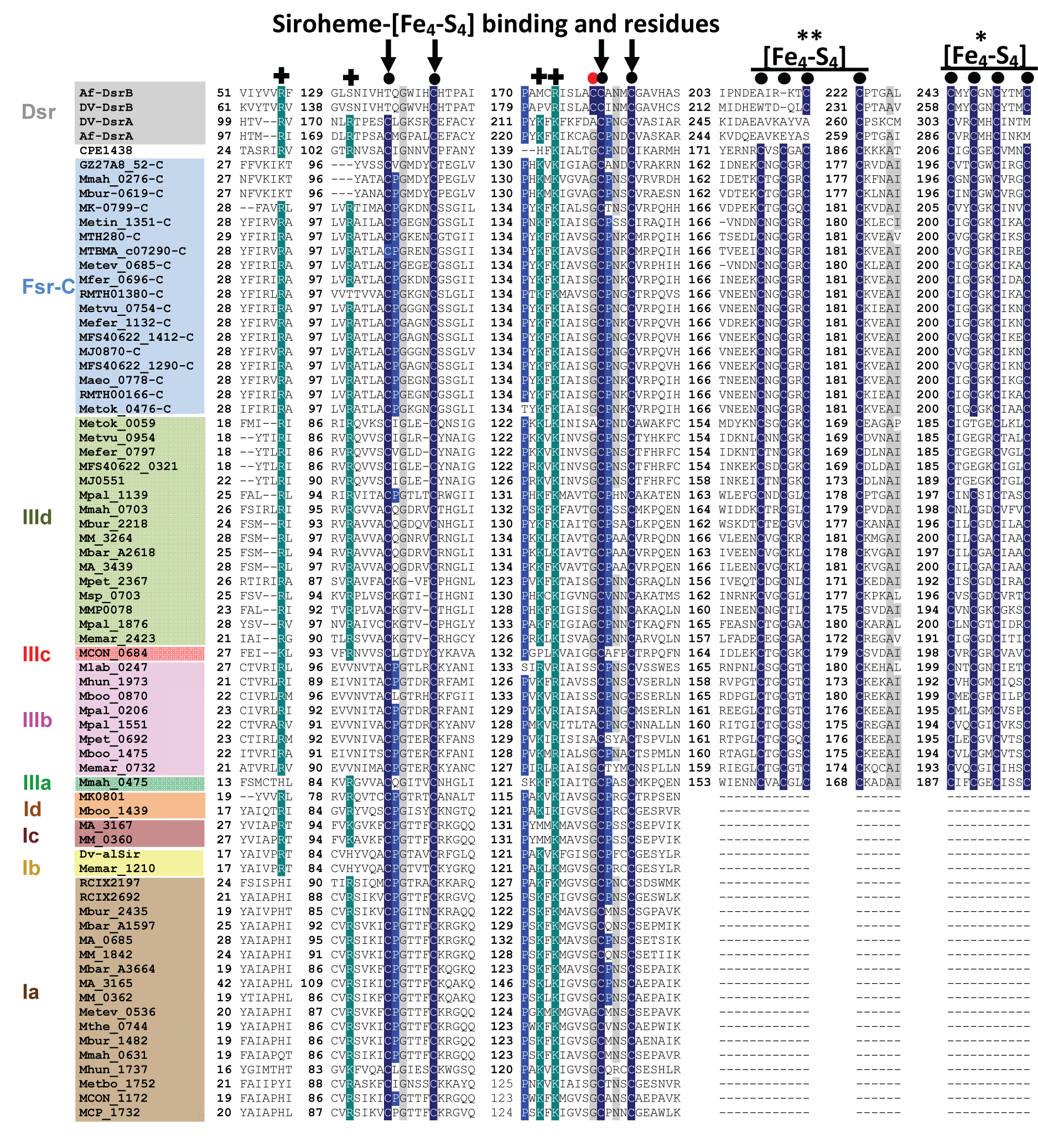

Supplement: Figure S1 — Primary structure comparison of Dsr-LPs of methanogenic archaea and archaeal and bacterial Dsr. Fsr-C, defined in Fig. 1. Structural type of Dsr-LP groups, as described in Fig. 3, shown as number Ia-d and IIIa-d left to the alignment (groups IIa-d, yet to be detected); “+”, sulfite binding Arg or Lys residues; arrows, residues involved in assembling [Fe4-S4]-coupled siroheme; over-line, sequence motif involved in assembling [Fe4-S4] cluster; * and **, peripheral and additional [Fe4-S4] centers, respectively. Black bullets, conserved cysteine residues for [Fe4-S4] and siroheme sites. Red bullet, non-conserved cysteine residues coupling [Fe4-S4] center with siroheme in Dv-DsrB. The details of the abbreviations for organism names are in the legend of Fig. 4. The following color shadings have been used to represent conserved residues: teal, arginine or lysine; dark blue, cysteine; blue, prolin; grey, other residues. The color shadings in the left panel representing various sulfite reductases correspond to the same in Fig. 4. (TIF) [file pone.0045313.s001.tif]

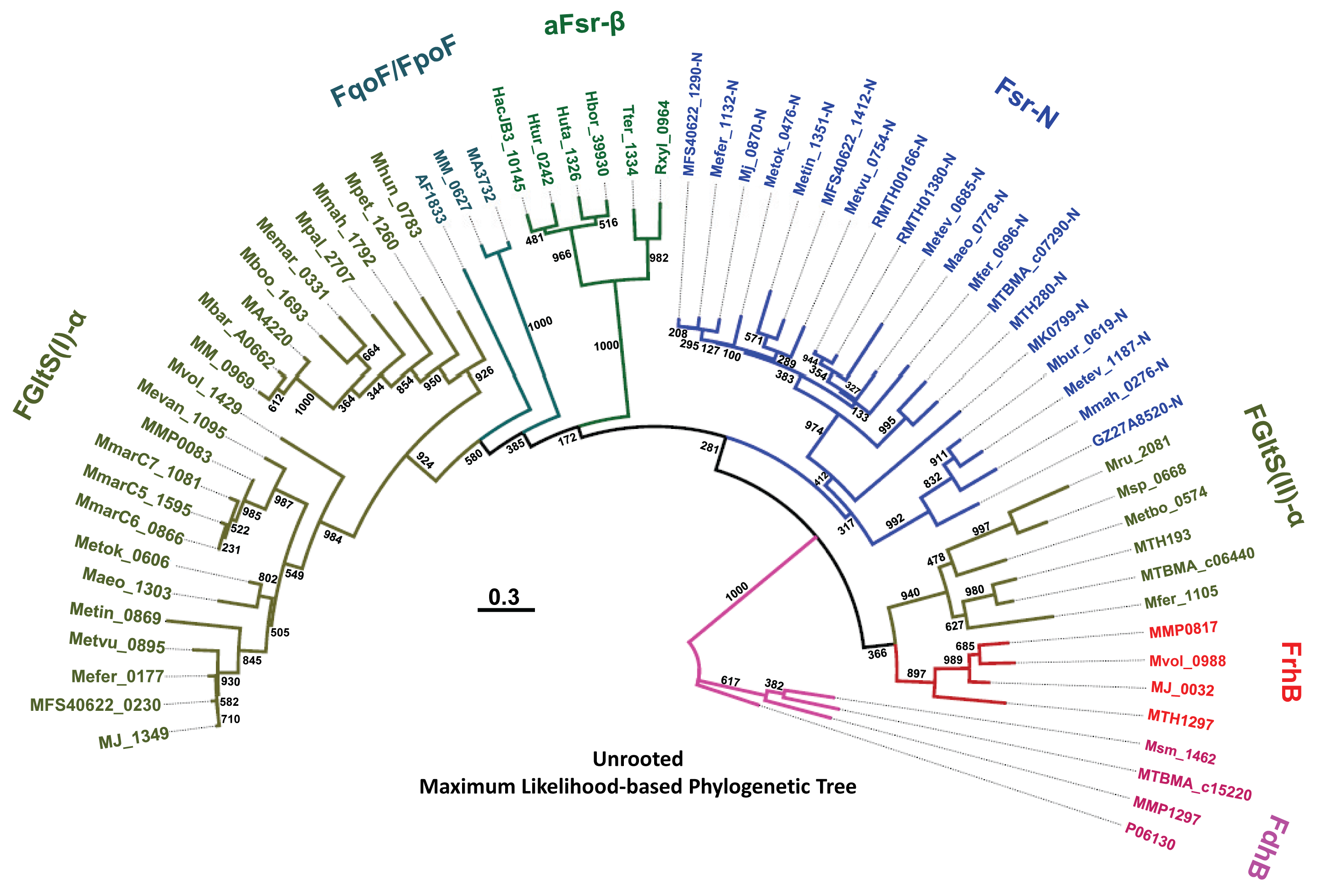

Supplement: Figure S2 — Phylogenetic tree of homologs of M. jannaschii Fsr-N according to Maximum Likelihood method. aFsr-β, FqoF, FpoF, and FGltS(I)-α are Fsr-N homologs. FrhB and FdhB are shown for comparison. See the legend of Fig. 1 for the full names of Fsr-N, aFsrβ, FqoF, FpoF and FGltS-α, and Fig. 6 for FrhB and FdhB. The ORF numbers followed by “-N”, Fsr-N homologs. Abbreviations for organism names preceding the ORF numbers (in addition to those described in Fig. 4 legend): Hbor, Halogeometricum borinquense DSM 11551; Huta, Halorhabdus utahensis DSM 12940; HacJB3, Halalkalicoccus jeotgali B3; Htur, Haloterrigena turkmenica DSM 5511; Rxyl_0964, Rubrobacter xylanophilus DSM 9941; Tter, Thermobaculum terrenum ATCC BAA-798; GZ27A8_52, uncultured archaeon related to Methanosarcina species; MmarC6, Methanococcus maripaludis C6; MmarC5, Methanococcus maripaludis C5; MmarC7, Methanococcus maripaludis C7; Mevan, Methanococcus vannielii SB; Mvol, Methanococcus voltae A3; AF, Archaeoglobus fulgidus DSM 4304, Msm, Methanobrevibacter smithii ATCC 35061; P06130, accession number for Methanobacterium formicicum FdhB. The bootstrap value shown at each branch is from 1000 replicates. Scale bar, number of amino acid substitutions per site. (TIF) [file pone.0045313.s002.tif]

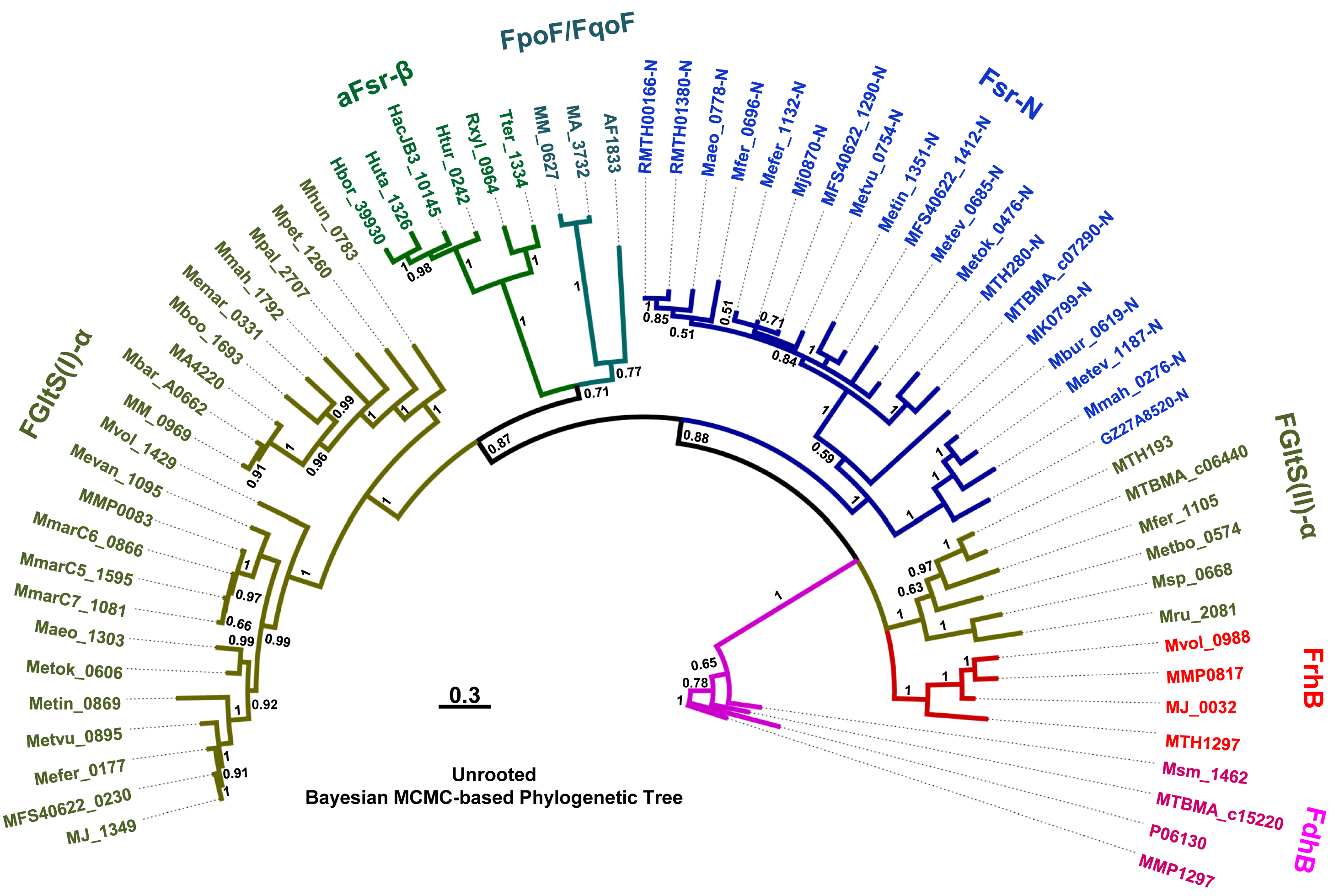

Supplement: Figure S3 — Phylogenetic tree of Fsr-N homologs based on Bayesian Markov chain Monte Carlo (MCMC) analysis. ORF numbers and all abbreviations used are described in the legend of Fig. S2. For each branch a posterior probability value (0–1) is shown. Scale bar, number of amino acid substitutions per site. (TIF) [file pone.0045313.s003.tif]

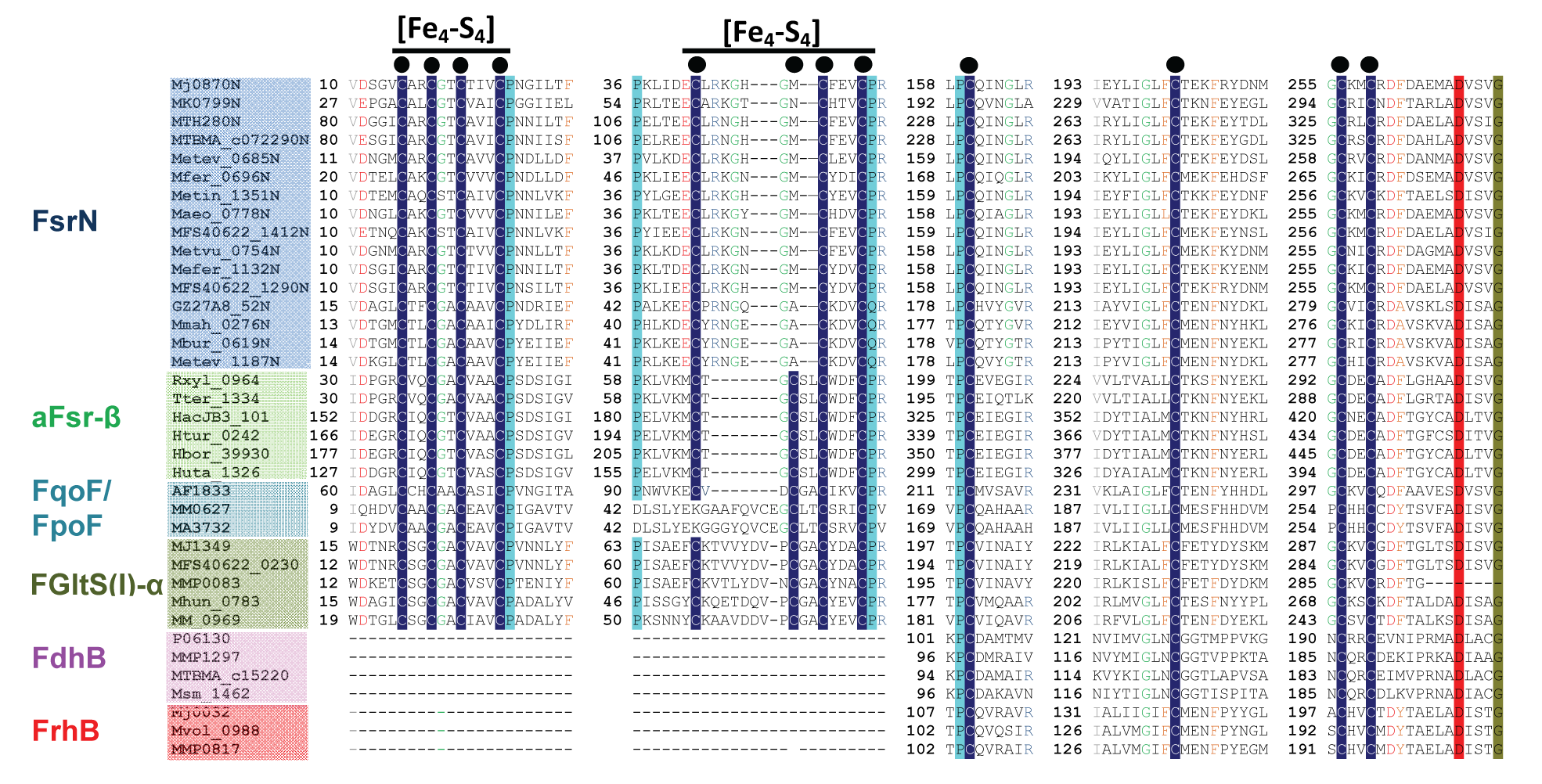

Supplement: Figure S4 — Primary structure comparison of homologs of M. jannaschii Fsr-N. aFsr-β, FqoF, FpoF and FGltS(I)-α are Fsr-N homologs. FGltS(II)-α, FrhB and FdhB are shown for comparison. The details of the abbreviation for ORF numbers are in the legend of Figs. 4 and S2. The color shadings and colored letters represent conserved and partially conserved amino acid residues, respectively: dark blue, cysteine; turquoise, prolin; red, aspartate and glutamate; green, glycine; orange, phenylalanine or tyrosine; grey, valine or isoleucine. Black bullets, conserved cysteine residues for [Fe4-S4] sites; over-line, sequence motif involved in assembling [Fe4-S4] cluster. The color shadings in the left panel representing various proteins correspond to the same in Figs. S2 and S3. (TIF) [file pone.0045313.s004.tif]
